# Supplementary material for: Type I Interferon Transcriptional Network Regulates Expression of Coinhibitory Receptors in Human T cells
Source: Res Sq. 2021 Jun 8:rs.3.rs-133494. Preprint. [Version 1] doi: 10.21203/rs.3.rs-133494/v1 (PMC8202434; doi:10.21203/rs.3.rs-133494/v1)

Supplementary Figure 1

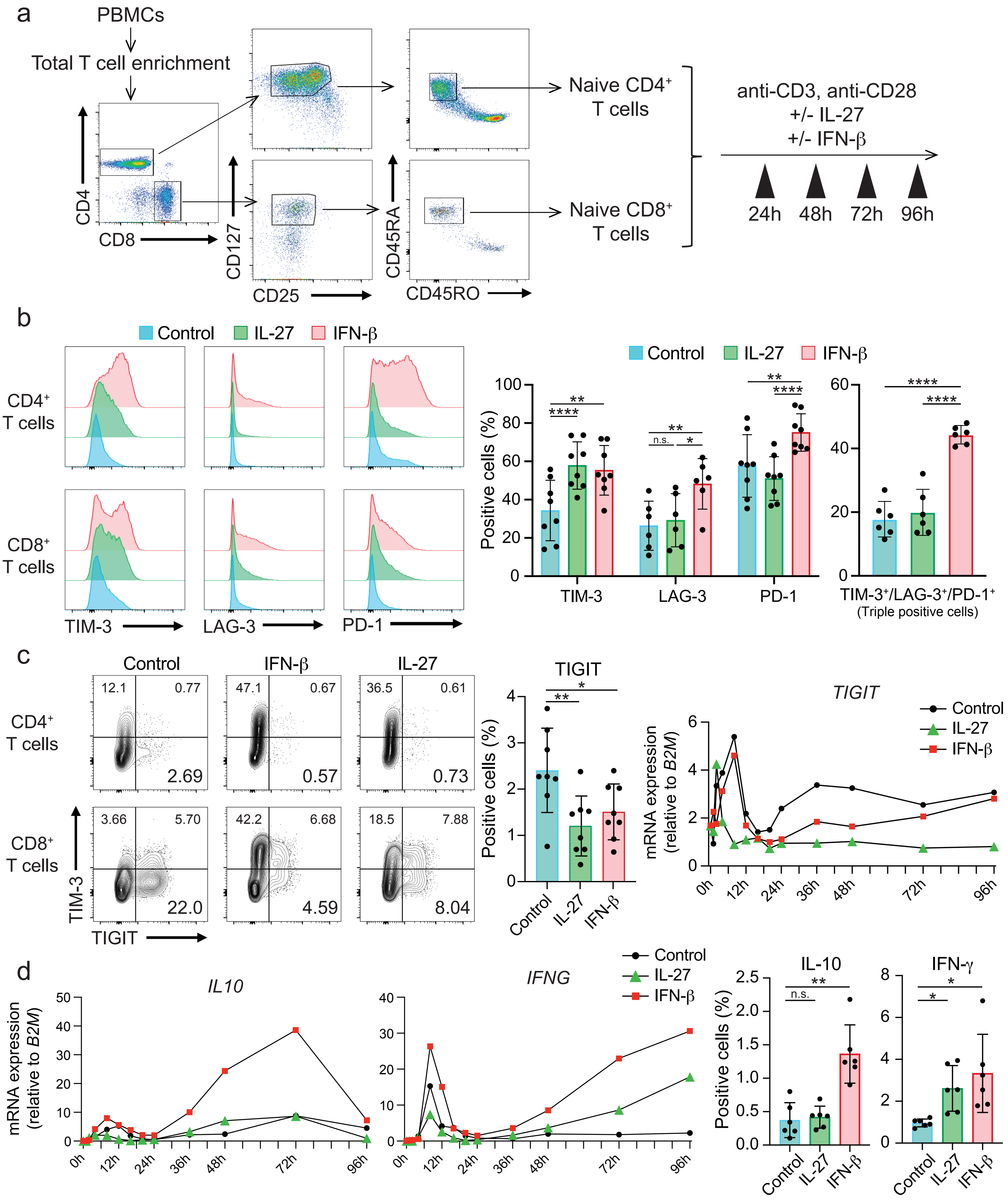

Supplementary Figure 2

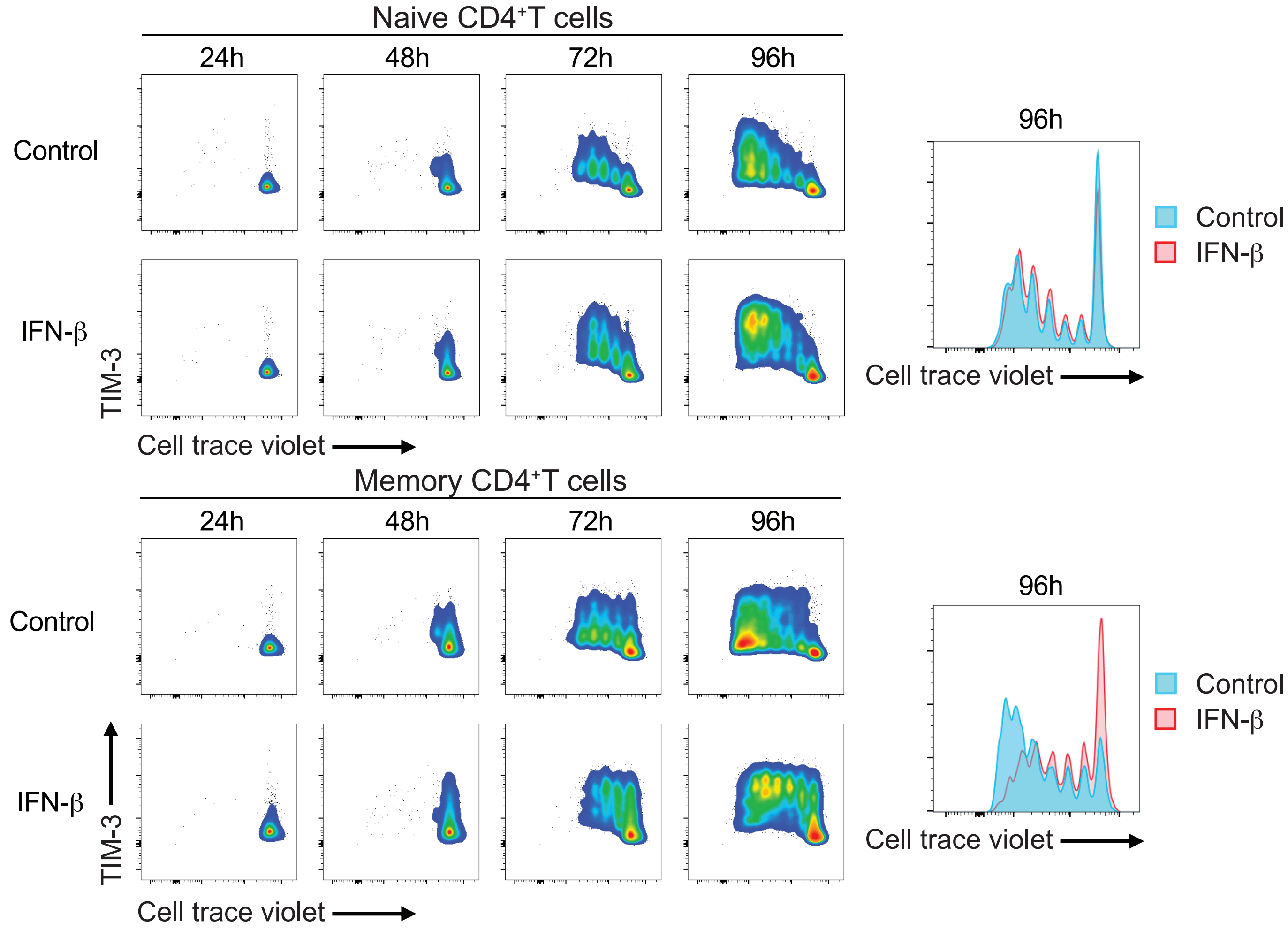

Supplementary Figure 3

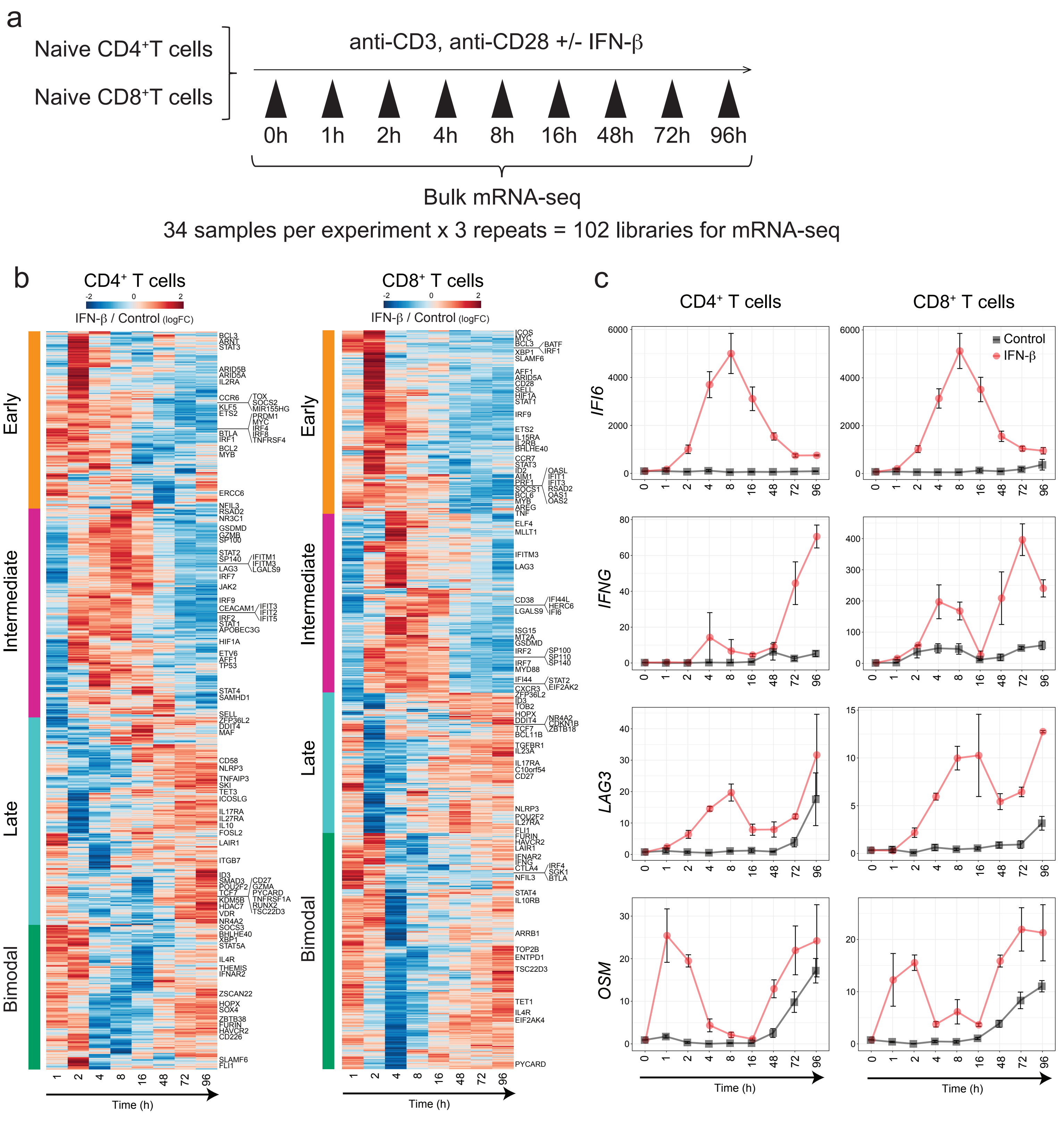

Supplementary Figure 4

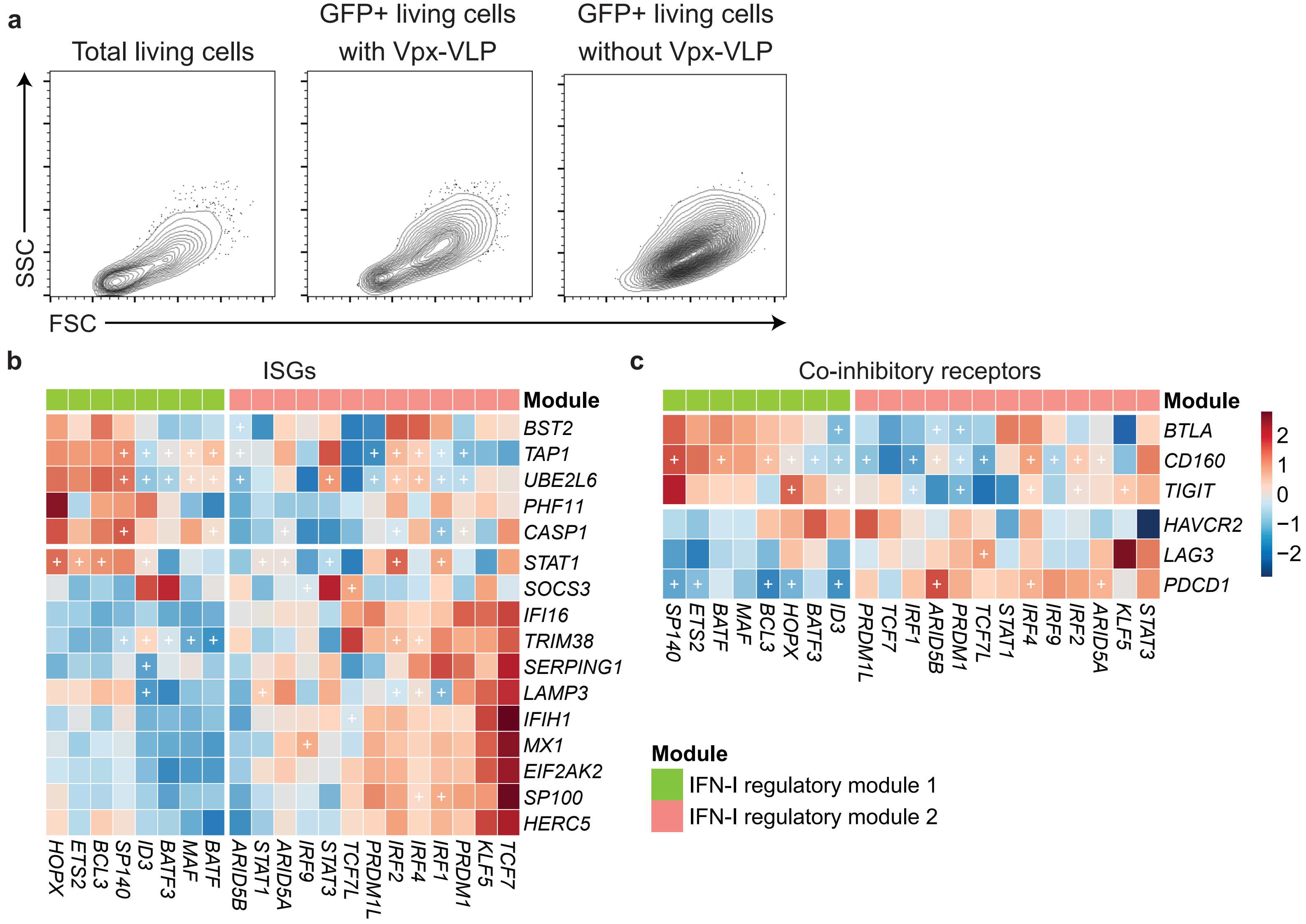

Supplementary Figure 5

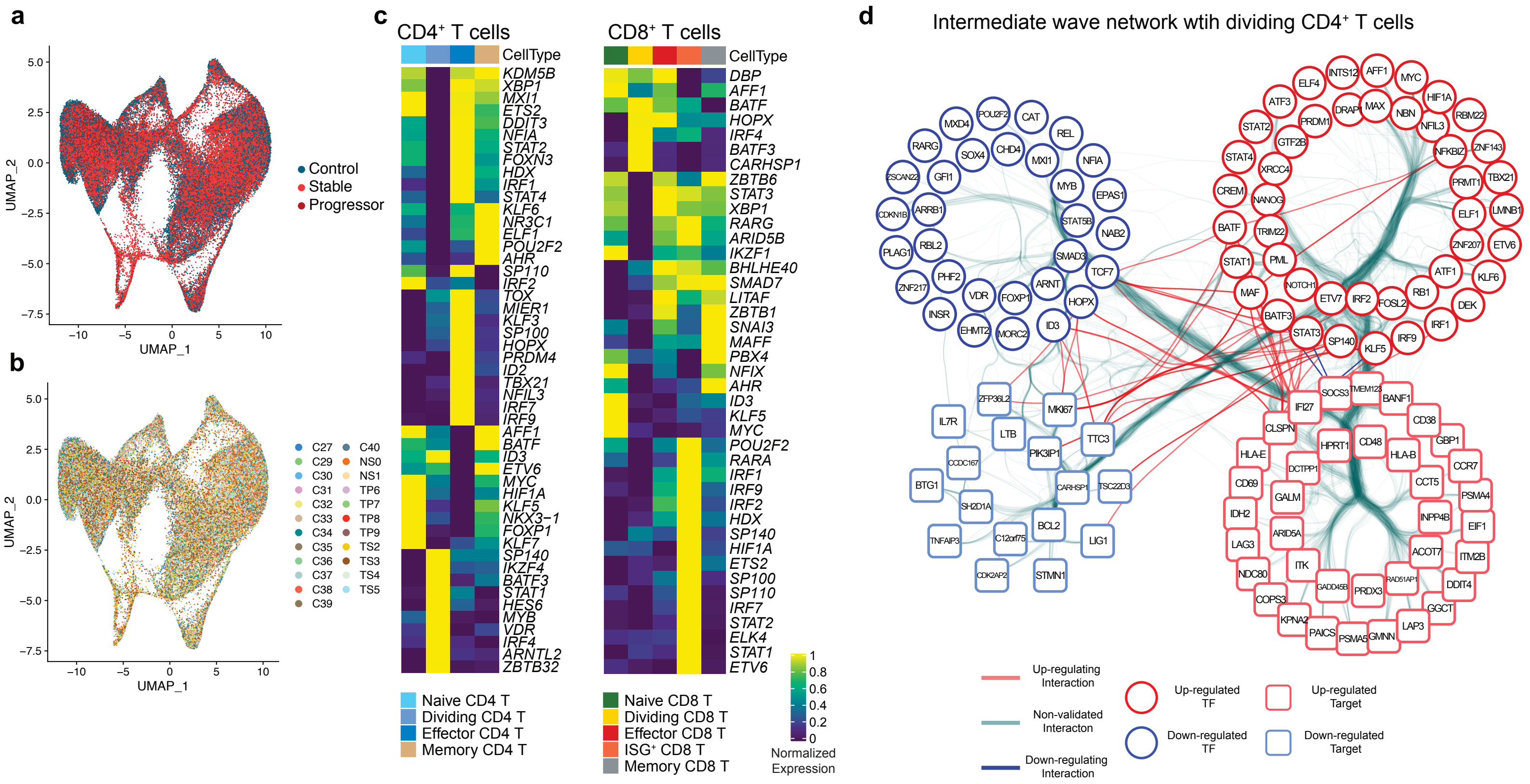

Supplement: Supplement 2 [file de3c017e066994bded23d029.pdf]
